# Effect of e-cigarette refill liquid on follicular development and estrogen secretion in rats

**DOI:** 10.18332/tid/146958

**Published:** 2022-04-08

**Authors:** Tairen Chen, Mengjing Wu, Yuting Dong, Bin Kong, Yufang Cai, Changchun Hei, Kai Wu, Chengjun Zhao, Qing Chang

**Affiliations:** 1 Key Laboratory of Fertility Preservation and Maintenance of Ministry of Education, School of Basic Medical Sciences, Ningxia Medical University, Yinchuan, China

**Keywords:** e-cigarette, ovary, granulosa cells, hippo signaling pathway, estrogen

## Abstract

**INTRODUCTION:**

Electronic cigarettes (e-cigarettes) have recently become popular as an alternative to conventional cigarettes. The aim of this study is to investigate the effects of e-cigarette refill liquid (e-liquid) on follicular development and estrogen secretion in rats and whether it is related to the Hippo signaling pathway, a pathway that can regulate follicle growth.

**METHODS:**

Ovaries from 21- and 35-day-old rats were divided into three groups: control (no intervention), 0.05 mg, and 0.5 mg (e-liquids containing 0.5 mg and 5 mg of nicotine/kg). The rates were cultured for three hours *in vitro*. At the end of culture, HE staining was performed to observe the follicle morphology and calculate the percentage of normal follicles, and the expression of Yes-associated protein (YAP, target factors of the Hippo signaling pathway) and CYP19 (aromatase, a key enzyme in estrogen synthesis) were observed by immunohistochemistry. Western blotting was performed to detect the expression levels of CYP19, YAP, phosphorylated YAP (PYAP), large tumor suppressor 2 (LATS2, factors upstream of YAP in the Hippo signaling pathway), and phosphorylated LATS2 (PLATS2). Estrogen concentrations were determined using ELISA.

**RESULTS:**

HE staining showed that the percentage of normal follicles decreased, and immunohistochemistry showed that the expression of CYP19 and YAP significantly decreased after e-liquid intervention. ELISA showed that the estrogen concentration in the ovaries decreased after e-liquid intervention. Western blot results indicated that CYP19, LATS2, and YAP expression, decreased after e-liquid intervention, but PLATS2 and PYAP expression increased.

**CONCLUSIONS:**

We found that the e-liquids may impair the development of rat ovarian follicles and reduce estrogen secretion through Hippo signaling pathway.

## INTRODUCTION

More than 480000 people in China die each year as a result of cigarette smoking, and cigarette smoke (CS) causes many undesirable effects on human health^1^. CS has been reported to cause follicle loss and induce autophagy in the ovaries^2,3^. The composition of CS is highly complex, with over 7000 chemicals generated during the combustion of tobacco, and exposure to this smoke and retention of smoke particles contribute to smoking-related injuries and diseases^4^. Nicotine, an important component of CS, can promote oxidative stress by producing reactive oxygen species (ROS) and has deleterious effects on blood cells and tissues^1^. Previous studies have reported that nicotine is addictive and can inhibit ovarian follicle development, by inducing apoptosis of granulosa cells (GCs), and inhibit estrogen synthesis^5,6^.

As an alternative to conventional cigarettes, electronic cigarettes (e-cigarettes) have attracted attention worldwide, mainly among the youth population, and have become a focus of public health concern^4,7^. However, there is a dearth of research on the public health hazards of e-cigarettes compared to those of traditional tobacco^8^. These electronic devices are designed to provide the sensation of conventional smoking without the harmful effects of conventional cigarettes. In the process of using e-cigarettes, e-cigarette refill liquids (e-liquids) are aerosolized. The main components of e-liquids are nicotine (optionally variable doses), vegetal glycerin (VG), propylene glycol (PG), and flavoring agents^9,10^. Moreover, in addition to aerosols generated after e-liquid heating, e-liquid can induce tissue oxidative damage and disrupt steroidogenesis^1,11^. Nonetheless, the effects of e-liquids on ovaries and the possible mechanisms remain unclear.

The Hippo signaling pathway consists of many conserved kinases whose main function is to regulate cell and tissue growth by affecting cell proliferation and apoptosis, which are important for ovarian tissue growth^12^. Yes-associated protein (YAP), a downstream factor of the Hippo signaling pathway, plays an important role in promoting GC proliferation, follicle development, and maintenance of normal ovarian function^13^. Knockdown of YAP in GCs reduces cytosolic aromatase (CYP19), which is a key enzyme for estrogen synthesis, resulting in a decrease in ovarian estrogen secretion^14,15^.

The Hippo signaling pathway and its downstream YAP are known to be associated with follicle growth, GC development, and estrogen secretion. Component of e-liquids, nicotine can inhibit follicle growth and estrogen synthesis^5,6^, and e-liquids without nicotine can cause tissue oxidative damage and disrupt steroidogenesis^1^. Therefore, this study was designed to investigate the effects of e-liquid on follicle development and estrogen secretion in rats, and whether they are related to the Hippo signaling pathway.

## METHODS

### Materials

*Animal*

A total of thirty-six 21-day-old (prepubertal) and thirty-six 35-day-old (puberty) female SD rats (SPF class, Laboratory Animal Center of Ningxia Medical University, Certificate of Conformity No: SCXK (Ning) 2020-0001) were housed at 22–24^o^C under 12 hours of light daily. The experiment strictly complied with the Guidelines for Ethical Review of Laboratory Animal Welfare issued by the General Administration of Quality Supervision, Inspection and Quarantine and the National Standardization Administration. The 21- and 35-day-old rats were divided into three groups, experiments were performed simultaneously and repeated at least three times.

*Main reagents*

Based on previous studies, we formulated e-liquids proportionally using nicotine salts (1.8%), VG (40%), PG (50%), distilled water (5%), and flavoring agents (3.2%)^1,10,11^. The nicotine salts (pro-salt-nix, 3118NS12-100MG), VG (PRB-297545V) and PG (PRB-362789P) were purchased from Probase (China) and the flavoring agents (tobacco flavor) were purchased from Proflavor (China).

The primary antibodies CYP19 (ab18995) and PYAP [Anti-YAP phospho (S127), ab76252] produced by Abcam (UK), YAP (DF3182) and LATS2 (DF7516) produced by Affbiotech (China), PLATS2 [Anti-phospho-LATS2(Ser83), bs-4082R] produced by BIOSS (China), and β-Actin (AC026) produced by Abclonal (China), were used. The secondary antibodies (HRP goat anti-rabbit IgG, AS014) produced by Abclonal (China), were used. A rabbit two-step immunohistochemistry kit (PV-9001) was provided by ZSGB-BIO (China). The ELISA rat estradiol assay kit (CEA461Ge) was purchased from Clould Clone Corp (China), the whole protein extraction kit (KGP2100) was provided by KeyGEN Bio TECH (China). Hematoxylin and eosin (AR1180) were from Boster Biological Technology (USA). A 4% paraformaldehyde (D16013) used, was produced by Saint-Bio (China). PBS Phosphate buffer used, was produced by Hyclones (USA). Sodium citrate antigen repair solution used, was produced by Boster Biological Technology (USA). BCA protein content test kit (KGP902) was provided by KeyGEN Bio TECH (China). SDS-PAGE Gel Kit (KGP113) was provided by KeyGEN Bio TECH (China). PVDF membrane was purchased from Millipore (USA). A 5% skim milk solution was provided by Thermofisher (USA). Xylene and absolute ethanol used, are domestic analytical pure reagents.

### Experimental methods

*Preparation of sample*

The medium was made according to F12:DMEM (1:1) (Gibco, USA) and contained 12% fetal bovine serum. The 21-day-old rats were identified as pre-pubertal, the 35-day-old rats were identified as pubertal, the estrous cycle was judged by vaginal smear, and were selected to perform the experiments at diestrus^16^.

The rats were sacrificed by cervical dislocation, the skin of the dorsal kidney region was disinfected with 75% alcohol, and then the ovaries were removed. Ovaries from 21- and 35-day-old rats were divided into three groups: control (no intervention) and 0.05 mg and 0.5 mg (e-liquids containing 0.5 and 5 mg of nicotine/kg). The rates were cultured for three hours *in vitro*. Methods of *in vitro* interventions of e-liquids refer to previous studies of nicotine *in vitro* interventions, and e-liquids analysis of cytotoxicity^8,17^. The concentration selection of the e-liquid was based on previous reports^1,10,11^. The ovaries of each group were incubated in a CO2 incubator at a constant temperature of 37°C for 3 h. The incubation and intervention times were referenced to previous experiments^18^, after incubation, they were rinsed three times with PBS to terminate the intervention. After the end of the intervention, one side of the ovaries was fixed in 4% paraformaldehyde solution for 12 h for morphological and immunohistochemical studies, and other side of the ovaries was stored at -80^o^C for Western blot and ELISA studies.

*Morphological observation*

After fixation, the ovaries were routinely dehydrated, paraffin-embedded, and serially sectioned at 5 μm intervals, with one section selected at every 6 intervals. The sections of ovaries were stained using HE staining, and then the morphology of ovaries was observed under microscope. The judgement criteria of healthy and atretic follicles were referred to previous reports^19,20^. The total number of follicles and the number of normal follicles in each group were counted. The above experiment was repeated at least three times.

*Immunohistochemical experiments*

Paraffin sections of ovaries were routinely dewaxed and hydrated, and the antigens were repaired by high pressure thermal retrieval method. The sections were placed in an autoclave with citrate buffer, heated and boiled for 15 minutes and then cooled naturally. Endogenous peroxidase blocker was added for 10 min at room temperature, PBS was rinsed and primary antibodies YAP (1:200) and CYP19 (1:200) were added dropwise and incubated for 60 min at 37°C. Subsequently, we added reaction enhancer for 20 minutes at room temperature, rinsed three times with PBS and added dropwise enhanced enzyme-labeled goat anti-rabbit IgG polymer and incubated for 20 minutes at room temperature, then added DAB chromogenic solution and allow to develop for five minutes at room temperature. Next, hematoxylin was re-stained for one minute, alcohol fractionation in hydrochloric acid for three seconds followed by rinsing to return blue for eight minutes, then gradient alcohol dehydration, xylene transparency for 15 minutes, neutral resin sealing, and reading. Finally, the expression of YAP and CYP19 was observed under microscopes, and the integral optical density (IOD) and the cumulative optical density on the immunohistochemical staining positive area (brown yellow) of each section were analyzed using image J software. Means and standard deviations were taken for statistical analysis. The above experiment was repeated at least three times.

*Western blot*

Ovarian tissue proteins were extracted using a protein extraction kit, and the protein sample concentrations were determined by the BAC method, and the protein concentration was then equalized. Electrophoresis was performed on a 10% SDS-PAGE gel with 8 μL (40 μg protein amount) of sample per well, and the proteins were transferred to PVDF membranes after electrophoresis. The membrane was blocked with 5% skim milk solution for 2 hours and incubated with primary antibodies against CYP19 (1:1000), YAP (1:1000), PYAP (1:1000), LATS2 (1:1000), PLATS2 (1:1000) and β-actin (1:50000) overnight at 4°C. The secondary antibodies (1:5000) were added the next day and incubated for one hour at room temperature. Using the ECL method, PVDF membrane was incubated in luminescent liquid (Super signal, Thermo,USA) for 1 minute. Then the bands were revealed by Chemiluminescence imager (AI600, GE, USA) by its automatic program. Finally, the band intensity was measured using Image J Software, with β-actin as the internal reference. The above experiment was repeated at least three times.

*Estradiol concentration was determined by ELISA*

After the culture, the ovaries were weighed and placed in a grinding tube with protein lysis solution (Lysis buffer, KGP2100, KeyGEN Bio TECH, China) at 1:10 and ground into a tissue homogenate in a freeze grinder, centrifuged at 12000g for 8 min, the precipitate was discarded, and the supernatant was used to determine the concentration of estradiol using ELISA estradiol assay kit. The above experiment was repeated at least three times.

### Statistical analysis

All experiment data are shown as mean ± SD and analyzed using analysis of variance (ANOVA), followed by Fisher’s Least Significant Difference Test (Fisher LSD) using the SPSS software. Statistical differences were considered significant when p<0.05.

## RESULTS

### Morphological observations and percentage of normal follicles

**Figure 1:**
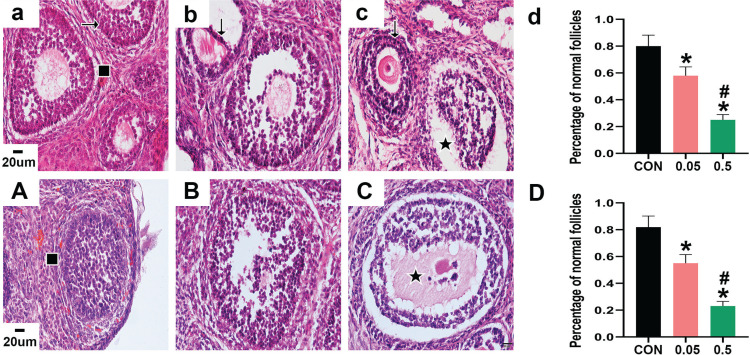
HE staining of ovarian sections observed under 40X microscope. Pictures a, b, c are the control, 0.05 mg, and 0.5 mg groups of 21-day-old rat ovaries. Picture d is the percentage of normal follicles in each group above. Pictures A, B, C are the control, 0.05 mg, and 0.5 mg groups of 35-day-old rat ovaries. Picture D is the percentage of normal follicles in each group above. → are normal GCs, ↓ are GCs nuclear pyknosis, ∎ are normal follicles, ★ are atretic follicles. Bar=20 μm. *Each group was compared with control group, p<0.05. # 0.5 mg compared with 0.05 mg group, p<0.05.

HE staining showed that the morphology of follicles was generally good in the control group of 21- and 35-day-old rat ovaries. The GC layer was intact and continuous, and the theca layer was clear, healthy, and intact. Nuclear pyknosis was occasionally observed in a small number of GCs. However, in the ovaries of the 21- and 35-day-old rats in the 0.05 mg group, GCs showed increased pyknosis and were disorganized, and not closely aligned. Oocytes of 21- and 35-day-old rat ovaries in the 0.5 mg group were damaged, and a large number of GCs showed nuclear pyknosis, with unclear nuclei and cytoplasm. The theca layer also appeared abnormal, and some were separated from the GC layer. Compared with the control group, the percentage of normal follicles in the 21- and 35-day-old rat ovaries was decreased in the 0.05 mg and 0.5 mg groups (p<0.05, Figure 1).

### Immunohistochemistry

**Figure 2:**
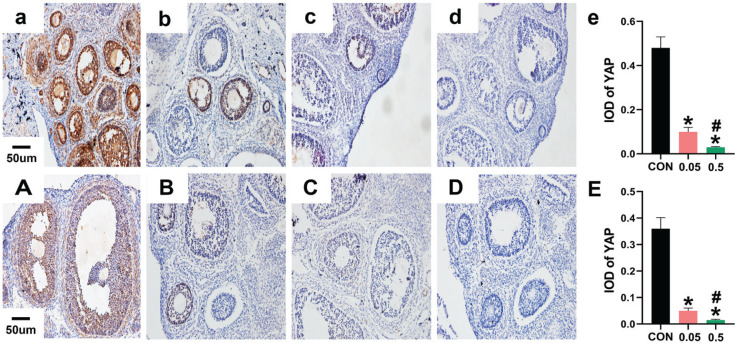
Immunohistochemical staining for YAP expression were observed under 20X microscope. Pictures a, b, c, d are the control, 0.05 mg, 0.5 mg and negative control groups of 21-day-old rat ovaries; Picture e is the integral optical density (IOD) of YAP expression in control, 0.05mg and 0.5mg groups. Pictures A, B, C, D are the control, 0.05 mg, 0.5 mg and negative control groups of 35-day-old rat ovaries. Picture E is the IOD of YAP expression in control, 0.05 mg and 0.5 mg groups. Bar=20 μm. *Each group was compared with control group, p<0.05. # 0.5 mg compared with 0.05 mg group, p<0.05.

**Figure 3:**
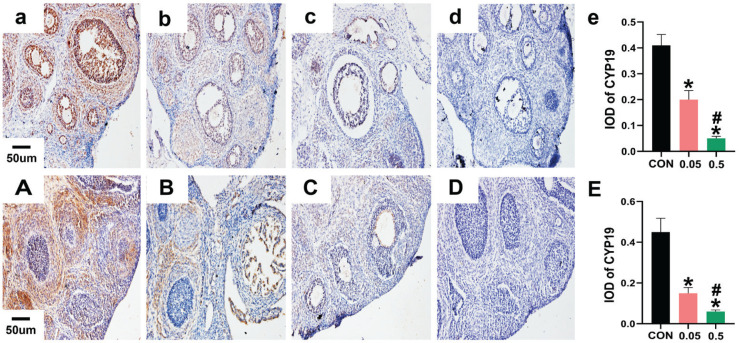
Immunohistochemical staining for CYP19 expression were observed under 20X microscope. Pictures a, b, c, d are the control, 0.05 mg, 0.5 mg and negative control groups of 21-day-old rat ovaries. Picture e is the integral optical density (IOD) of CYP19 expression in control, 0.05 mg and 0.5 mg groups. Pictures A, B, C, D are the control, 0.05 mg, 0.5 mg and negative control groups of 35-day-old rat ovaries. Picture E is the IOD of CYP19 expression in control, 0.05 mg and 0.5 mg groups. Bar=20 μm. *Each group was compared with control group, p<0.05. # 0.5 mg compared with 0.05 mg group, p<0.05.

In the control group of the 21- and 35-day-old rat ovaries, YAP was predominantly expressed in the nucleus of follicular GCs as small brown particles, with a small amount in theca cells and interstitial cells. Compared with the control group, in both the 21- and 35-day-old rat ovaries, YAP (IOD) of ovaries was decreased in the 0.05 mg and 0.5 mg groups (Figure 2). In the 21- and 35-day-old rat ovaries of the control group, CYP19 was located in follicular GCs, theca cells, and interstitial cells. Compared with the control group, in both the 21- and 35-day-old rat ovaries, CYP19 (IOD) of ovaries was decreased in the 0.05 mg and 0.5 mg groups (p<0.05, Figure 3).

### Western blot

**Figure 4:**
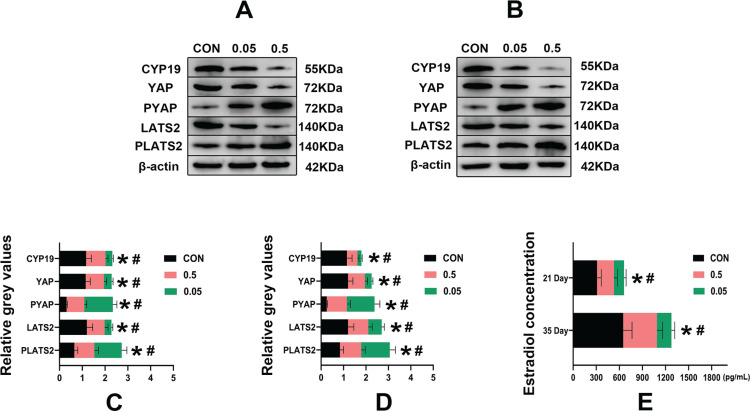
Western blot analysis of control, 0.05 mg and 0.5 mg groups of 21- (A) and 35-day-old (B) rat ovaries. Pictures C, D are the relative grey values analysis of CYP19, YAP, PYAP, LATS2, PLATS2 in each group compared with β-actin. Picture C is 21-day-old rat ovaries and D is 35-day-old rat ovaries. Picture E is the estrogen concentration (pg/mL) in each group of 21- and 35-day-old rat ovaries determined by ELISA. *Each group was compared with its control group, p<0.05. # 0.5 mg compared with 0.05 mg group, p<0.05.

Compared with the control group, in both the 21- and 35-day-old rat ovaries, the relative grey values of CYP19, LATS2 and YAP in the 0.05 mg and 0.5 mg groups were decreased (p<0.05). In contrast, PLATS2 and PYAP were increased in the 0.05 mg and 0.5 mg groups, (p<0.05) (Figure 4).

### ELISA ovarian estradiol concentration measurement

Compared with the control group, in both the 21- and 35-day-old rat ovaries, the estradiol concentration was decreased in the 0.05 mg and 0.5 mg groups (p<0.05) (Figure 4 E).

## DISCUSSION

The toxic effects of e-cigarettes are multifaceted, and in addition to the obvious harm to the respiratory and cardiovascular systems, the effects on other systems of the body are gradually attracting attention^21-23^. Preliminary evidence suggests that e-cigarettes cause damage to the female reproductive system^24,25^.

Morphological observations by hematoxylin and eosin staining (Figure 1) showed that the morphology of follicles in 21- and 35-day-old rat ovaries changed significantly after e-liquid intervention, and the percentage of normal follicles decreased, while GC nuclear pyknosis increased. The results indicate that the e-liquids could disrupt the normal development of follicles in both 21- and 35-day-old rat ovaries by inducing nuclear pyknosis in GCs. Previous studies also found that nicotine, one of the constituents of e-liquid, can cause ovarian damage and granulosa cell apoptosis, and that e-liquid can cause oxidative damage and cytotoxicity^1,5,8,17^.

Immunohistochemistry results showed that YAP in the 21- and 35-day-old rat ovaries, predominantly in follicular GCs, appeared to decrease after e-liquid intervention (Figure 2). YAP, an important factor in the Hippo signaling pathway, functions mainly by entering the nucleus to bind to TEAD, activating transcriptional function, and promoting cell and tissue growth^13,26^. Thus, e-liquids significantly inhibited YAP in rat follicular GCs, indicating a negative regulatory effect of e-liquids on follicle growth.

Immunohistochemistry showed that CYP19 was distributed in the follicular GCs, theca cells, and interstitial cells in the 21- and 35-day-old rat ovaries and decreased after e-liquid intervention (Figure 3). CYP19 is a key enzyme in estrogen synthesis^15^; therefore, the decrease in CYP19 indicates that e-liquids have an inhibitory effect on estrogen secretion in the ovaries. Previous studies have also reported that nicotine, one of the constituents of e-liquids, can inhibit CYP19 activity, and e-liquids (without nicotine) can also disrupt steroidogenesis^6,11^.

Western blot results showed that the e-liquids could inhibit CYP19, LATS2, and YAP and upregulate PLATS2 and PYAP in the 21- and 35-day-old rat ovaries (Figure 4). LATS2 and YAP are both factors in the Hippo signaling pathway. LATS2 is upstream of YAP, and when the Hippo signaling pathway is activated, a series of phosphorylation reactions occur, causing a decrease in LATS2 and an increase in PLATS2, which further leads to YAP being phosphorylated (PYAP, inactivated state) and stagnated in the cytoplasm. This leads to a reduction in non-phosphorylated YAP (YAP, activated state), which inhibits GC growth and estrogen synthesis. Conversely, increased YAP has a promoting effect^12,14,27^.

These results indicate that e-liquids increase the phosphorylation levels of LATS2 and YAP in the Hippo signaling pathway, leading to a decrease in YAP expression in 21- and 35-day-old rat ovaries. Interestingly, when YAP expression increases, CYP19 also increases, and vice versa, implying that the regulation of CYP19 is associated with YAP and regulated by the Hippo signaling pathway.

ELISA results (Figure 4 E) showed a significant decrease in estradiol concentration in 21- and 35-day-old rat ovaries after e-liquid intervention, which might be related to the inhibition of YAP by e-liquids. When YAP is increased, CYP19 is also increased, and vice versa. CYP19 is a key enzyme for the synthesis of estrogen^15^. It has also been previously reported that knockdown of YAP in GCSs could reduce CYP19 expression^14^.

### Limitations

Some limitations of our study must be considered. We used e-liquids, so it cannot be determined whether the ovarian damage found was caused by nicotine or other components in e-liquids. The present results demonstrated only the effects of e-liquids on the ovaries of young rats. We plan to conduct studies on the effects of single components of e-liquids on ovaries.

## CONCLUSIONS

The present study found that e-liquids can impair follicle development and estrogen secretion of ovaries through activating the Hippo signaling pathway.

## Data Availability

The data supporting this research are available from the authors on reasonable request.
